# Clinical Utility of Rapid Exome Sequencing Combined With Mitochondrial DNA Sequencing in Critically Ill Pediatric Patients With Suspected Genetic Disorders

**DOI:** 10.3389/fgene.2021.725259

**Published:** 2021-08-19

**Authors:** Xuejun Ouyang, Yu Zhang, Lijuan Zhang, Jixuan Luo, Ting Zhang, Hui Hu, Lin Liu, Lieqiang Zhong, Shaoying Zeng, Pingyi Xu, Zhenjiang Bai, Lee-Jun Wong, Jing Wang, Chunli Wang, Bin Wang, Victor Wei Zhang

**Affiliations:** ^1^Department of Pediatrics, Zhujiang Hospital, Southern Medical University, Guangzhou, China; ^2^Department of Gastroenterology, Shanghai Children’s Hospital, Shanghai, China; ^3^Department of Vasculocardiology, Guangdong Provincial People’s Hospital, Guangzhou, China; ^4^Department of Neurology, First Affiliated Hospital of Guangzhou Medical University, Guangzhou, China; ^5^Department of Critical Care Medicine, Children’s Hospital of Soochow University, Suzhou, China; ^6^Department of Human and Molecular Genetics, Baylor College of Medicine, Houston, TX, United States; ^7^AmCare Genomics Lab, Guangzhou, China

**Keywords:** rapid exome sequencing, mitochondrial diseases, mtDNA sequencing, pediatric patients, genetic disorders

## Abstract

Genetic disorders are a frequent cause of hospitalization, morbidity and mortality in pediatric patients, especially in the neonatal or pediatric intensive care unit (NICU/PICU). In recent years, rapid genome-wide sequencing (exome or whole genome sequencing) has been applied in the NICU/PICU. However, mtDNA sequencing is not routinely available in rapid genetic diagnosis programs, which may fail to diagnose mtDNA mutation-associated diseases. Herein, we explored the clinical utility of rapid exome sequencing combined with mtDNA sequencing in critically ill pediatric patients with suspected genetic disorders. Rapid clinical exome sequencing (CES) was performed as a first-tier test in 40 critically ill pediatric patients (aged from 6 days to 15 years) with suspected genetic conditions. Blood samples were also collected from the parents for trio analysis. Twenty-six patients presented with neuromuscular abnormalities or other systemic abnormalities, suggestive of suspected mitochondrial diseases or the necessity for a differential diagnosis of other diseases, underwent rapid mtDNA sequencing concurrently. A diagnosis was made in 18 patients (45.0%, 18/40); three cases with *de novo* autosomal dominant variants, ten cases with homozygous or compound heterozygous variants, three cases with hemizygous variants inherited from mother, three cases with heterozygous variants inherited from either parent, and one case with a mtDNA mutation. The 18 patients were diagnosed with metabolic (*n* = 7), immunodeficiency (*n* = 4), cardiovascular (*n* = 2), neuromuscular (*n* = 2) disorders, and others. Genetic testing reports were generated with a median time of 5 days (range, 3–9 days). Thirteen patients that were diagnosed had an available medical treatment and resulted in a positive outcome. We propose that rapid exome sequencing combined with mitochondrial DNA sequencing should be available to patients with suspected mitochondrial diseases or undefined clinical features necessary for making a differential diagnosis of other diseases.

## Introduction

Genetic disorders are the leading cause of hospitalization, morbidity and mortality in pediatric patients, especially in the neonatal or pediatric intensive care unit (NICU/PICU) (Stevenson and Carey, 2004; Willig et al., 2015; French et al., 2019). A genetic diagnosis in the critical illness remains a great challenge for the physicians when the patients’ phenotypes has not fully been expressed due to age to make a clear clinical diagnosis. Moreover, many traditional diagnostic methods are too slow to provide clinically useful information to physicians. However, an early and accurate genetic diagnosis would be of great value for the clinical management of the patient and family (Australian Genomics Health Alliance Acute Care Flagship, Lunke et al., 2020; Cakici et al., 2020; Dimmock et al., 2020). For those genetic diseases that can be treated, rapid diagnosis can provide timely interventions and avoid other unnecessary or potentially harmful therapies, thus reducing mortality and intensive care. For genetic diseases currently lacking effective treatments, the diagnosis may be useful in evaluating prognosis and preventing possible complications, as well as facilitating further genetic counseling for the family.

The recent development of rapid genome-wide sequencing (exome or whole genome sequencing, ES or WGS) has been widely used for genetic analyses in critically ill pediatric patients with suspected genetic disorders in the PICU/NICU (Saunders et al., 2012; Petrikin et al., 2015, 2018; Kingsmore et al., 2019). Generally, rapid exome sequencing (WES or gene panels) has delivered a molecular diagnosis for patients with the turnaround time (TAT) of 1–2 weeks, and positively changed the medical management (Elliott et al., 2019; Gubbels et al., 2020; Smigiel et al., 2020). The molecular diagnosis applied by rapid WGS was generally completed less than one week, even within 24 h, potentially revolutionizing clinical practice (Clark et al., 2019; Wang et al., 2020). ES focuses on targeted sequencing of the protein-coding regions of the nuclear genome, while WGS involves sequencing the entire genome including coding regions, intergenic regions and the mitochondrial genome. Previous studies reported that most of the patients identified as genetic conditions by rapid ES or WGS were monogenic disorders, and just a few cases who underwent WGS were diagnosed with a mitochondrial disorder caused by a single nucleotide variant (SNV) in mitochondrial DNA (mtDNA) (Petrikin et al., 2015; Willig et al., 2015).

Mitochondrial disorders are a group of complicated and heterogeneous diseases caused by mutations in genes that lead to defective oxidative phosphorylation (OXPHOS) and ATP synthesis (Wortmann et al., 2017). The diagnosis of mitochondrial disorders has always been challenging due to the clinical features being variable. Although the central nervous system (CNS) is most often involved, other single organ systems or multiple organ systems may be affected, such as muscle, liver, heart, kidneys, gastrointestinal tract, endocrine glands, and others (Kisler et al., 2010; Schon et al., 2020). Therefore, mitochondrial disorders enter the differential diagnosis of many common diseases. Mitochondrial diseases with childhood onset often present in the newborn period, however, it is very difficult to make an early diagnosis because the clinical phenotypes overlap with other common disorders (Tulinius and Oldfors, 2011; Honzik et al., 2012). The multimeric protein complexes comprising the OXPHOS system have a dual genetic origin (nuclear DNA or mtDNA), while the majority of subunits are encoded by the nuclear genome that can be identified effectively by using ES or WGS. However, mitochondrial genome sequencing is not routinely available in rapid genetic diagnosis programs, which may fail to diagnose mtDNA mutation-associated diseases.

In this study, we explored the clinical utility of combined rapid clinical exome sequencing (CES) and mtDNA sequencing in 40 critically ill pediatric patients with suspected genetic disorders. The CES in this study was a uniquely designed sub-exome panel comprised of approximately 5000 known genes causing Mendelian diseases, while whole mtDNA sequencing was performed using long-PCR followed by next generation sequencing (NGS).

## Methods

### Patient Cohort

**FIGURE 1:**
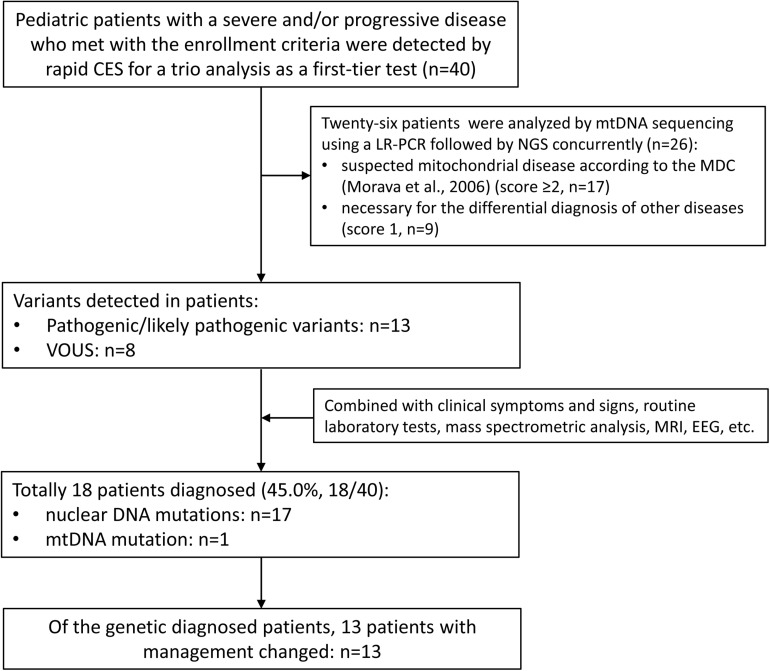
The flowchart of the genetic diagnosis in the 40 pediatric patients. CES, clinical exome sequencing; MDC, mitochondrial disease criteria; VOUS, variants of unknown significance.

This multi-center study was performed at five hospitals in China from June 2018 to May 2020, which was approved by the Ethical Review Committee of the hospitals. The enrollment criteria were as follows: (1) pediatric patients with a severe and/or progressive disease but without a definite diagnosis, such as metabolic disturbances, neurological abnormalities, immune system defects, respiratory and/or cardiovascular failure and multiple congenital anomalies; (2) written informed consent was obtained from both parents prior to genetic testing; (3) both parents were available, and rapid CES was applied as a first-tier genetic test for a trio analysis. A total of 40 patients were enrolled in this study, including 29 patients in PICU/NICU and 11 patients in the other pediatric wards (Table 1). Twenty-six patients with neuromuscular abnormalities or other systemic abnormalities underwent rapid mtDNA sequencing concurrently. The flowchart of this study was shown in Figure 1.

**TABLE 1:** General description of the 40 pediatric patients with rapid genetic diagnosis.

Characteristic	Total cases (*n* = 40)	Diagnosed by genetic analysis (*n* = 18)
**Sex**		
Male	24 (60.0%, 24/40)	11 (61.1%, 11/18)
Female	16 (40.0%, 16/40)	7 (38.9%, 7/18)
**Age at genetic testing (1 d∼15 y)**		
Neonates: Age < 28 d	6 (15.0%, 6/40)	4 (22.2%, 4/18)
Infants: 28 d ≤ Age < 1 y	25 (62.5%, 25/40)	12 (66.7%, 12/18)
Children: Age ≥ 1 y	9 (22.5%, 9/40)	2 (11.1%, 2/18)
**Patients from departments**		
Neonatal intensive care unit (NICU)	18 (45.0%, 18/40)	7 (36.8%, 7/18)
Pediatric intensive care unit (PICU)	11 (27.5%, 11/40)	7 (36.8%, 7/18)
Other pediatric wards	11 (27.5%, 11/40)	4 (21.1%, 4/18)
**Classification based on clinical phenotypes at genetic testing**		
Neuromuscular diseases	5 (12.5%, 5/40)	4 (22.2%, 4/18)
Digestive diseases	4 (10.0%, 4/40)	0
Cardiovascular diseases	4 (10.0%, 4/40)	2 (11.1%, 2/18)
Endocrine diseases	3 (7.5%, 3/40)	2 (11.1%, 2/18)
Metabolic-related diseases	3 (7.5%, 3/40)	3 (16.7%, 3/18)
Immunodeficiency diseases	2 (5.0%, 2/40)	1 (5.6%, 1/18)
Multi-system diseases	19 (47.5%, 19/40)	6 (33.3%, 6/18)

*CES, clinical exome sequencing; mtDNA, mitochondrial DNA; d, day; y, year.*

### Clinical Exome Sequencing

Peripheral blood samples of the patients and their parents were collected and the genomic DNA was extracted using the SolPure Blood DNA kit (Magen) according to the manufacturer’s instructions. The Q800R Sonicator (Qsonica) was used to disrupt the genomic DNA to generate 300–500 bp fragments. The paired-end libraries were prepared following the Illumina library preparation protocol. Custom-designed NimbleGen SeqCap probes (Roche NimbleGen, Madison, WI) were used for in-solution hybridization to enrich target sequences, which included ∼5000 genes associated with known Mendelian genetic diseases based on databases of Online Mendelian Inheritance in Man (OMIM), Human Gene Mutation Database (HGMD), and literature (AmCare Genomic Laboratory, Guangzhou, China). The NextSeq500 or NovaSeq 6000 sequencer (Illumina, San Diego, CA) was used for sequencing of enriched DNA. The average coverage depth was 200×, and at least 98% of the target regions were covered by 20×. The CES technology is capable of detecting various of known variant types, including SNVs, copy number variations (CNVs), small insertion and deletions (indels), chromosome aneuploidy, uniparental disomy (UPD), and mosaicism (Feng et al., 2015; Yu et al., 2016).

### Variant Analysis and Interpretation

Raw-image data conversion and demultiplexing were performed following Illumina’s primary data analysis pipeline using CASAVA version 2.0 (Illumina). Low-quality reads (Phred score <Q20) were removed before demultiplexing. Sequences were aligned to the hg19 reference genome by NextGENe (SoftGenetics, State College, PA) using the recommended standard settings for single-nucleotide variant and insertion/deletion discovery. The variants with minor allele frequency (MAF) >1% in the Asian population were filtered out. Variant annotation was further confirmed through literature and population databases, including 1000 Genomes, dbSNP, GnomAD, Clinvar, HGMD, and OMIM. Genetic evaluation of pathogenicity of candidate gene variants was performed using multiple computational algorithms, including SIFT (Craig Venter Institute), Polyphen-2 (Harvard University), and MutationTaster (NeuroCure Cluster of Excellence). The interpretation of variants was performed according to the American College of Medical Genetics (ACMG) guidelines (Richards et al., 2015).

### mtDNA Sequencing

The whole mitochondrial genome sequencing (16,569 bp) was performed using long-PCR followed by next generation sequencing (Yang et al., 2020). The mtDNA was extracted from peripheral blood from patients and maternal samples using a commercial DNA extraction kit (Mike Bio, China) according to the manufacturer’s instructions. The whole mitochondrial genome was amplified using long-range PCR, and disrupted by sonication using a Q800R Sonicator (Qsonica). High throughput sequencing was performed using a NextSeq 500 sequencer (Illumina, United States). The average depth of sequencing was at least 5000×. After the data was obtained, the bioinformatics analysis of the gene sequence was performed to confirm the locus information of the pathogenic genes. This assay detects single nucleotide variants with heteroplasmy >2%.

## Results

### Clinical Characteristics

The clinical characteristics of the patients who went through the rapid CES and mtDNA sequencing were summarized in Table 1. Our cohort comprised of 24 males and 16 females with a median age of 4.5 months (range, 6 days–11 years) at the time rapid CES was performed, including 6 newborns less than one month old, 25 infants aged 1–12 months old, and 9 young children aged 1–11 years old. Among the patients, there were 18 patients in the NICU, 11 patients in the PICU, and 11 patients in other pediatric wards. Regarding the family history, 2 patients were twin sisters with the same phenotype (patient ID: P14, P15). All the patients were born to non-consanguineous parents, and none had an affected parent.

### Genetic Diagnosis, TAT

According to the genetic test results and clinical phenotypes, a genetic diagnosis was made in 18 patients (18/40, 45.0%); three cases with *de novo* autosomal dominant variants (involving the genes *KRAS*, *SCN5A*, and *WT1*), nine cases with homozygous or compound heterozygous variants (involving the genes *IL10RA*, *CD19*, *SLC37A4*, *UPB1*, *GCDH*, *AMPD1*, *VWF*, and *LHX3*), three cases with hemizygous variants inherited from mother (including the genes *HSD17B10*, *SLC9A6*, and *NAA10*), two cases with heterozygous variants inherited from either parent (*ABCC8* and *MYH7* genes), and one case with a mtDNA mutation (*MT-ND6* gene) (Table 2). The variation types detected in the 18 patients included missense and frameshift mutations, while zero clinically significant CNVs were detected in this study.

**TABLE 2:** Clinical features and genetic outcome of the 18 patients diagnosed.

Cases diagnosed in the category	Patient ID	Sex/Age at testing	Clinical features	Gene/OMIM ID	Ref. Seq (hg19)	Variants/Transcript_ID	Variant classifi- cation	Clinically reported (Yes/No)	Zygosity	Inheri- tance pattern	Disease
**Metabolic-related diseases: *n* = 6**	P8	M/3m	Mass spectrometry showed multiple metabolite abnormalities, hyperlactacidemia	*SLC37A4* (602671)	11:118898518	c.446G > A (p.G149E) [NM_001164278]	LP	Yes	Hom	AR; Inherited from both parents	Glycogen storage disease type Ic (MIM: 232240)
	P13^#^	M/18m	Psychomotor retardation, developmental regression at 10 months old, strabismus, nystagmus, bucking, feeding difficulties, hypotonia	*MT-ND6*	M:14430	m.14430A > G (p.W82R) [NC_012920]	LP	Yes	mtDNA mutation load of 91.3%	Maternal inheritance (7.9%)	Leigh syndrome (MIM: 256000)
	P24	M/1m	Respiratory distress syndrome, mass spectrometry showed elevated β-UP and β-UIB	*UPB1* (606673)	22:24919647	c.977G > A (p.R326Q) [NM_016327]	LP	Yes	Hom	AR; Inherited from both parents	Beta-ureidopropionase deficiency (MIM: 613161)
	P28^#^	M/7d	Fetal macrosomia, hypoglycemia	*ABCC8* (600509)	11:17428474	c.3250_3252 delins13 (p.T1042Qfs ^∗^ 75) [NM_000352]	LP	No	Het	AD/AR; Inherited from father	Familial hyperinsulinemic hypoglycemia (MIM: 256450)
	P36^#^	M/11m	Sudden irritability and dyspnea, hyperlactacidemia, respiratory and circulatory failure	*HSD17B10* (300256)	X:53458838	c.503A > G (p.Y168C) [NM_004493]	VOUS	No	Hem	XL; Inherited from mother	HSD10 mitochondrial disease (MIM: 300438)
	P38^#^	F/6m	Obnubilation, hypotonia, infantile encephalopathy, brain MRI showed abnormal signals in bilateral basal ganglia and white matter, and atrophy of the temporal lobes	*GCDH* (608801)	19:13004345; 19:13007744	c.383G > A (p.R128Q); c.873delC (p.N291Kfs ^∗^ 41) [NM_000159]	LP; LP	Yes; No	Compound Het	AR; Inherited from both parents	Glutaricaciduria type I (MIM: 231670)
**Immunodefi- ciency diseases: *n* = 4**	P1	F/7m	Diarrhea, perianal abscess, pyrexia, skin eczema	*IL10RA* (146933)	11:117860269	c.301C > T (p.R101W) [NM_001558]	LP	Yes	Hom	AR; Inherited from both parents	Early-onset inflammatory bowel disease (MIM: 613148)
	P5	F/10m	Diarrhea, pyrexia, splenomegaly, rash, hyperbilirubinemia	*KRAS* (190070)	12:25378647	c.351A > C (p.K117N) [NM_033360]	LP	No	Het	AD; *De novo*	RAS-associated autoimmune leukoproliferative disorder (MIM: 614470)
	P14^#^ and P15^#^ (twins)	F/6m	Severe infection, electrolyte disturbance, severe metabolic alkalosis	*CD19* (107265)	16:28943883; 16:28947515	c.305C > (p.P102R); c.988A > G (p.T330A) [NM_001178098]	VOUS; VOUS	No; No	Compound Het	AR; Inherited from both parents	Common variable immunodeficiency (MIM: 613493)
**Cardiovascular diseases: *n* = 2**	P18^#^	M/5y	Malignant arrhythmia: ventricular tachycardia, ventricular fibrillation, atrial tachycardia, atrial fibrillation	*SCN5A* (600163)	3:38592971	c.4892G > A (p.G1631D) [NM_001099404]	LP	Yes	Het	AD; *De novo*	Familial atrial fibrillation (MIM: 614022)
	P21^#^	F/4m	Poor appetite, enlarged left ventricle, reduced systolic and diastolic function	*MYH7* (160760)	14:23902427	c.211G > (p.V71M) [NM_000257]	LP	No	Het	AD; Inherited from mother	Cardiomyopathy dilated type 1S (MIM: 613426)
**Neuromuscular diseases: *n* = 2**	P23^#^	M/9m	Motor retardation, hypotonia and weakness of the whole body	*AMPD1* (102770)	1:115223070; 1:115220125	c.676G > A (p.D226N); c.1334C > T (p.A445V) [NM_000036]	VOUS; VOUS	No; No	Compound Het	AR; Inherited from both parents	Myoadenylate deaminase deficiency myopathy (MIM: 615511)
	P26^#^	M/2m	Seizures, head CT showed bilateral symmetrical low-density images in parietal lobe	*SLC9A6* (300231)	X:135080641	c.604A > G (p.R202G) [NM_001042537]	VOUS	No	Hem	XL; Inherited from mother	Christianson syndrome (MIM: 300243)
**Multi-system diseases: *n* = 2**	P16^#^	M/2m	Small for gestational age infant, cyanoderma, hyoxemia, hyperbilirubinemia, special face	*NAA10* (300013)	X:153199841	c.109T > C (p.S37P) [NM_003491]	LP	Yes	Hem	XL; Inherited from mother	Ogden syndrome (MIM: 300855)
	P39	M/23d	Low response, hypospadias, short penis, systemic pitting edema	*WT1* (607102)	11:32413565	c.1385G > A (p.R462Q) [NM_024426]	LP	Yes	Het	AD; *De novo*	Denys-Drash syndrome (MIM: 194080)
**Hematological diseases: *n* = 1**	P20^#^	F/6d	Respiratory distress, thrombocytopenia, coagulation abnormalities	*VWF* (613160)	12:6132811; 12:6103733	c.3365C > T (p.T1122M); c.6104G > A (p.G2035D) [NM_000552]	LP; LP	Yes; Yes	Compound Het	AR; Inherited from both parents	Von Willebrand disease type 3 (MIM: 277480)
**Endocrine diseases: *n* = 1**	P22^#^	M/11d	Small head circumference, hypoglycemia, hypothyroidism, left cryptorchidism, deafness	*LHX3* (600577)	9:139090825; 9:139090762	c.550T > C (p.S184P); c.613G > C (p.V205L) [NM_014564]	LP; LP	No; No	Compound Het	AR; Inherited from both parents	Combined pituitary hormone deficiency type 3 (MIM: 221750)

*^#^, CES and mtDNA sequencing; M, male; F, female; d, day; m, month; y, year; LP, Likely pathogenic; VOUS, various of unknown significance; Hom, homozygote; Het, heterozygote; Hem, hemizygote; mtDNA, mitochondrial DNA; AR, autosomal recessive; AD, autosomal dominant; XL, X-linked.*

The rapid trio CES combined with mtDNA sequencing strategy enabled us to obtain a median TAT of 5 days from blood sample receipt to test reporting to ordering clinician (range 3–9 days). In three severely ill patients, the TAT of rapid CES and mtDNA sequencing was 3 days. For *de novo* variants or uncertain variants that suspected homologous interference needed to be confirmed by Sanger sequencing, thus, the final report was issued 2 days thereafter.

### Impact of the Rapid Genetic Diagnosis on Clinical Procedures

Thirteen patients that were diagnosed had an available medical treatment option and resulted in a positive outcome. For example, a female infant (ID: P38) was admitted to the PICU at 6 months of age with irritability, dystonia, and atrophy of the temporal lobes. MRI results showed abnormal bilateral basal ganglia and white matter signal, and atrophy of the temporal lobes. The rapid CES and mtDNA sequencing were applied to the patient concurrently, and test reports were issued within 5 days. CES identified compound heterozygous mutations (p.R128Q and p.N291Kfs ^∗^ 41) in the *GCDH* gene (OMIM: 608801), then the patient was diagnosed with glutaric aciduria type I. She was immediately treated by a low-lysine diet and carnitine supplementation according to the guidelines for the management of glutaric aciduria type I (Heringer et al., 2010) and were considered for further long-term follow-up as indicated.

A male infant (ID: P8) was admitted to the PICU at 3 months of age with lactic acidosis and hypoglycemia. A molecular diagnosis of glycogen storage disease Ib caused by compound heterozygous mutations (p.G149E and p.G149E) in the *SLC37A4* gene (OMIM: 602671) was detected by rapid CES. He was immediately treated by continuous night-time intragastric glucose infusion through a nasogastric tube, frequent feedings during the daytime with high complexity carbohydrates, and oral administration of uncooked cornstarch was also recommended after 6 months old according to the guidelines for the management of glycogen storage disease type I (Kishnani et al., 2014). These treatments successfully made the boy maintain a normal blood glucose concentration and corrected most of the other metabolic abnormalities.

Patient 18 (ID: P18) was admitted to the PICU at 4 years old with malignant arrhythmia including ventricular tachycardia, ventricular fibrillation, atrial tachycardia and atrial fibrillation. A *de novo* heterozygous mutation (p.G1631D) in the *SCN5A* gene (OMIM: 600163) was detected by rapid CES. The *SCN5A* gene defects have been associated with Brugada syndrome, familial atrial or ventricular fibrillation, and long QT syndrome. He was given beta blockers (propranolol), and cessation of symptoms was observed.

Patient 28 (ID: P28) was admitted to the NICU at birth with hypoglycemia, hyperinsulinism, and adrenal hyperplasia. He underwent rapid CES and mtDNA sequencing, and a diagnosis of “familial hyper insulinemic hypoglycemia” was made by detecting a heterozygous mutation (p.T1042Qfs ^∗^ 75) in the *ABCC8* gene (OMIM: 600509) inherited from the father. He was given diazoxide therapy combined with frequent feedings, and showed an excellent response to this treatment with substantially improved plasma glucose concentrations.

## Discussion

Of the known genetic diseases according to the database of OMIM, the majority predominantly affect infants and children, which are the leading cause of infant mortality and pediatric hospital admissions. There is evidence that a significant number of genetic diseases present with clinical phenotypes during the first 28 days or the first year of life. However, precise early diagnosis of genetic diseases in these pediatric patients is very challenging, because the full clinical symptoms may not be evident in newborns or infants. Many studies have shown that genome-wide sequencing based on NGS technologies are powerful approaches for genetic testing in clinical settings. In recent years, rapid WGS and WES have been effectively used for genetic analysis in critically ill pediatric patients with suspected genetic disorders in the PICU/NICU, with a diagnosis rate ranging from 30 to 72.2% in different pediatric patient cohorts (Bourchany et al., 2017; van Diemen et al., 2017; Farnaes et al., 2018; Brunelli et al., 2019; Elliott et al., 2019; Sanford et al., 2019; Smigiel et al., 2020; Wang et al., 2020). In the present study, we applied rapid CES in 40 patients with severe and/or progressive diseases (most in NICU/PICU), as well as mtDNA sequencing in 26 patients that could not exclude mitochondrial disorders. Among the 40 patients, 17 patients were diagnosed as monogenetic disorders caused by mutations on nuclear genome, and one patient was diagnosed as a mitochondrial disorder caused by a mtDNA mutation, with an overall yield rate of 45.0% (18/40).

Inborn errors of metabolism (IEM) are a group of rare genetic diseases that generally result from impaired enzyme activities in specific metabolic pathways by genetic mutations. Recent articles reported more than 1015 known IEM, of which mitochondrial disorders are the most common group (about 23%) of inherited metabolic diseases (Ferreira et al., 2019; Ismail et al., 2019). To date, pathogenic variants in more than 300 nuclear genes and 37 mitochondrial genes are known to cause mitochondrial diseases (Schon et al., 2020). The genetic basis of mitochondrial disorders is very complex, and Mendelian inheritance and maternal inheritance patterns can all lead to both familial and sporadic cases. In this study, IEM was found in 6 patients, including two cases with mitochondrial disorders. A recent study on 18 unrelated infants in an ICU has also shown that mitochondrial disorders identified by rapid WES are the main cause of IEM (Smigiel et al., 2020). The prevalence of mtDNA mutation diseases is estimated to be at least 1 in 5000 (Skladal et al., 2003; Schaefer et al., 2004), and early-onset disease (<1 year) has been reported in many studies (Honzik et al., 2012; Bannwarth et al., 2013; Akesson et al., 2019; Imai-Okazaki et al., 2019). However, mtDNA sequencing is not routinely available in current rapid genetic diagnosis programs, which may fail to diagnose mtDNA mutation-associated diseases. In addition to nuclear genome, WGS can also provide a relatively good coverage (approximately 1000–2000×) of the mitochondrial genome to identify mtDNA variant, specifically a single nucleotide variant (Saunders et al., 2012; Petrikin et al., 2015; Brockhage et al., 2018; Husami et al., 2020; Riley et al., 2020), while ES may detect single nucleotide variants in mtDNA (Pronicka et al., 2016; Puusepp et al., 2018). The mitochondrial genome may be impacted by nuclear mitochondrial DNA segments (NUMTs), resulting in false positive mtDNA variants, failure to distinguish between heteroplasmy and homoplasmy, and inaccurate quantification of mtDNA variants (Ramos et al., 2013; Puertas and Gonzalez-Sanchez, 2020). In this study, the whole mitochondrial genome sequence was performed using a long-PCR based NGS method (Saunders et al., 2012; Yang et al., 2020). An 18-month-old male patient (ID: P13) with early-onset psychomotor retardation and developmental regression had a missense mutation (m.14430A > G, p.W82R) with mutation load of 91.3% in the *MT-ND6* gene that was diagnosed with Leigh syndrome.

The clinical diagnosis of mitochondrial diseases present challenges for physicians, starting with when and how to investigate a suspected mitochondrial disorder. When a patient is clinically suspected of mitochondrial disease, the traditional approach is to exclude other common or metabolic disorders followed testing affected tissue with biochemical and histochemical assays to reach a final diagnosis. However, this procedure is expensive, invasive, time-consuming, and often no definitive diagnosis is reached. The modified mitochondrial disease criteria (MDC) in 2006 classified patients as having a “possible,” “probable,” or “definite” mitochondrial disorder according to score based on clinical signs and symptoms, metabolic/imaging tests, and morphology (Morava et al., 2006). With the advent of next-generation sequencing, exome sequencing was used to identify genetic defects in suspected mitochondrial patients. Wortmann et al. reported that a molecular diagnosis was achieved in 57% of the subgroup of patients with the highest suspicion for a mitochondrial disease, while several genes associated with neuromuscular disorders were identified in the subgroup of patients with the lowest suspicion for a mitochondrial disorder (Wortmann et al., 2015). The application of WES in patients suspected with mitochondrial disease indicated that nuclear DNA variants are more common, single nucleotide variants in mtDNA may also be detected, and that a significant proportion of non-mitochondrial disorders caused by other variants were also found (Pronicka et al., 2016; Puusepp et al., 2018). In a multicenter cohort of 136 patients clinically diagnosed with mitochondrial disorders, mtDNA restriction fragment length polymorphism (RFLP) and/or exome sequencing (including WES or gene panels) were performed to confirm the genetic etiology, and the results showed that 51% had a maximal mitochondrial score (8/8) predicting a definite mitochondrial disorder, 33% had a probable mitochondrial disorder (5–7/8), and 16% had a possible mitochondrial disorder (2–4/8) (Witters et al., 2018). Previous studies indicated that WES analysis has been successfully implemented as a first-tier test for mitochondrial disorders as well as for the mimicking disorders in clinical practice, and clinical and biochemical phenotyping is essential for successful application of WES to diagnose these patients. In view of the broad phenotypic and genotypic heterogeneity of mitochondrial disorders, we suggest that exome sequencing in combination with mtDNA sequencing is applied to identify genetic defects for all patients that could not exclude a mitochondrial disorder according to the MDC.

Early diagnosis of suspected genetic disorders can help guide clinical management, greatly improve patient outcomes, and reduce health care cost. In this study, the average turnaround time to reach a diagnosis was 5 days, with a range of 3–9 days, which is significantly faster than routine exome sequencing. Currently, rapid whole exome and target sequencing can deliver a molecular diagnosis for patients with the TAT of 1–2 weeks, while the TAT of rWGS was generally less than one week even within 24 h, positively changing the medical management. In this study, 13 patients that were diagnosed had an available medical treatment and resulted in a positive outcome. We have not analyzed the economic benefits and issue, but it is highly possible that rapid CES and mtDNA sequencing as a first-tier test is time and cost-effective by significantly shortened the time of hospitalization for patients in NICU/PICU.

In conclusion, rapid CES proved to be an effective diagnostic tool for critically ill pediatric patients with suspected genetic disorders, which prevented a range of unnecessary investigations and guided the appropriate treatment. We have demonstrated the feasibility and utility of incorporating rapid mitochondrial genome sequencing combined with ES in patients whose diagnosis or differential diagnosis includes mitochondrial disorders.

## Data Availability Statement

The datasets for this article are not publicly available due to concerns regarding participant/patient anonymity. Requests to access the datasets should be directed to the corresponding authors.

## Ethics Statement

The studies involving human participants were reviewed and approved by The ethics committee of Zhujiang Hospital. Written informed consent was obtained from the individual(s)’ and minor(s)’ legal guardian/next of kin, for the publication of any potentially identifiable images or data included in this article.

## Author Contributions

XO, BW, and VZ designed the study and drafted the initial manuscript. YZ, LjZ, JL, TZ, HH, LL, LqZ, SZ, PX, ZB, and CW supervised all aspects of data collection, analysis, and manuscript production. L-JW and JW reviewed and revised the manuscript. All authors reviewed and revised the manuscript and approved the final manuscript as submitted.

## Conflict of Interest

JW, CW, and VZ were employed by AmCare Genomics Lab. The remaining authors declare that the research was conducted in the absence of any commercial or financial relationships that could be construed as a potential conflict of interest.

## Publisher’s Note

All claims expressed in this article are solely those of the authors and do not necessarily represent those of their affiliated organizations, or those of the publisher, the editors and the reviewers. Any product that may be evaluated in this article, or claim that may be made by its manufacturer, is not guaranteed or endorsed by the publisher.
